# Supplementary material for: Asynchronous magnetic resonance elastography: Shear wave speed reconstruction using noise correlation of incoherent waves
Source: Magn Reson Med. 2022 Oct 27;89(3):990–1001. doi: 10.1002/mrm.29502 (PMC9792433; doi:10.1002/mrm.29502)
Supplement: Supplementary file 1 — DATA S1 MATLAB code used to generate the numerical simulations of Figure 1 [file MRM-89-990-s002.zip › k-Wave/helpfiles/mendousse.html]

mendousse :: Functions (k-Wave)


# mendousse

Compute Mendousse's solution for nonlinear wave propagation in viscous media.

## Syntax

```
p_mendousse = mendousse(x, t, source_freq, p0, c0, rho0, BonA, alpha_0)
```

## Description

`mendousse` calculates the propagation of a monofrequency plane wave source in a thermoviscous medium with absorption given by `alpha_0*f^2`. The solution is taken from Eq. (264) in Chapter 4 of Nonlinear Acoustics by Hamilton and Blackstock (2008). The infinite sum is adaptively truncated when the moving average of the previous five sum terms is smaller than a predefined convergence percentage (0.01 percent by default). An example of using the function is given below.

```
% define the properties used in the simulation
source_freq = 1e6;      % [Hz]
p0 = 5e6;               % [Pa]
c0 = 1500;              % [m/s]
rho0 = 1000;            % [kg/m^3]
BonA = 12;
alpha_0 = 0.5;          % [dB/(MHz^2 cm)]
x = 0:75e-6:40e-3;      % [m]
t = x ./ c0;            % [s]

% compute solution
p_mendousse = mendousse(x, t, source_freq, p0, c0, rho0, BonA, alpha_0);

% plot
figure;
plot(x * 1e3, p_mendousse * 1e-6);
ylabel('Pressure [MPa]');
xlabel('Distance [mm]');
scaleFig(1, 0.65);
```

## Inputs

|  |  |
| --- | --- |
| `x` | position [m] |
| `t` | time [s] |
| `source_freq` | frequency of plane wave [Hz] |
| `p0` | source pressure [Pa] |
| `c0` | medium sound speed [m/s] |
| `rho0` | medium density [kg/m^3] |
| `BonA` | nonlinearity parameter B/A |
| `alpha_0` | absorption coefficient [dB/(MHz^2 cm)] |

## Outputs

|  |  |
| --- | --- |
| `p_mendousse` | calculated pressure field [Pa] |

## Examples

- Modelling Nonlinear Wave Propagation

## See Also

`focusedBowlONeil`
